# Editorial: Surgical techniques for the management of pain

**DOI:** 10.3389/fpain.2023.1120174

**Published:** 2023-02-13

**Authors:** Anthony K. Allam, Ashwin Viswanathan, Jason M. Schwalb, Parag G. Patil, M. Benjamin Larkin

**Affiliations:** ^1^ School of Medicine, Baylor College of Medicine, Houston, TX, United States; ^2^ Department of Neurosurgery, Baylor College of Medicine, Houston, TX, United States; ^3^ Department of Neurosurgery, University of Texas, MD Anderson, Houston, TX, United States; ^4^ Department of Neurosurgery, Henry Ford Medical Group, West Bloomfield, MI, United States; ^5^ Department of Neurosurgery, University of Michigan Medical School, Ann Arbor, MI, United States

**Keywords:** ablative surgery, chronic pain, cancer pain, neuropathic pain, neuromodulation

Editorial on the Research TopicSurgical techniques for the management of pain

Chronic pain has reached epidemic proportions in the US, with an estimated 20% of the US population affected, more than cancer, heart disease, and diabetes combined (1, 2). Systematic reviews have linked chronic pain to anxiety, depression, and reduced quality of life, with the associated economic costs reaching up to $635 billion per year (3–6). Despite the magnitude of the problem, treating medically intractable chronic pain, including those patients with cancer-related pain, remains inadequate. While effective for acute pain, pharmacological therapy either fails to address chronic pain adequately or results in adverse effects, including gastritis, increased risk of pulmonary or cardiovascular complications, and addiction (7). When medical management proves unsuccessful or has undesired side effects, surgical approaches can help manage pain.

Surgical interventions with the primary goal of pain management in patients with medically intractable chronic pain or cancer pain can be divided into ablation and neuromodulation procedures. Ablative procedures aim to interrupt or destroy target pathways that either conduct pain or result in the sensation of pain (8). These targets include the dorsal root ganglion, the spinothalamic tract, the thalamus, and the anterior cingulate cortex. On the other hand, neuromodulation techniques aim to reduce pain through invasive or non-invasive modulation of neural signals in cortical and sensory pathways. Examples include spinal cord and deep brain stimulation. While neuromodulation procedures are often preferred due to their reversibility and adjustability, ablative procedures have simpler postoperative management and no long-term hardware-associated risks. In this Research Topic, we have compiled two review articles, 1 case report, and 1 case series exploring various ablative and neuromodulation techniques for managing chronic pain.

Spinal cord stimulation is a powerful tool to help alleviate neuropathic pain cost-effectively cheaper than medical management and primary spine surgery (9). One of the most successful neuromodulation procedures for pain management is spinal cord stimulation (SCS). In this procedure, SCS leads are implanted adjacent to the dorsal columns, delivering electrical impulses to reduce pain sensation. Initial stimulation profiles were tonic and induced paresthesias in the affected area; however, later advances allowed for intermittent stimulation in the form of high-frequency and burst stimulation without stimulation-induced paresthesias. Although early researchers hypothesized that the gate theory of pain was the primary mechanism of action, the continued efficacy of SCS, despite varying stimulation profiles, paints a more complex and nuanced picture. Sheldon et al. reviewed the effectiveness of SCS and briefly discussed dorsal root ganglion stimulation (DRGS) for the management of neuropathic pain (Figure 1A). In randomized controlled trials, the authors found high-frequency and burst stimulation superior to tonic stimulation. They further discussed the future of closed-loop SCS, where the intensity of stimulation is automatically adjusted to deliver the best pain-free outcomes. Current neurofeedback mechanisms for closed-loop SCS include accelerometry data and evoked compound action potentials.

**Figure 1:**
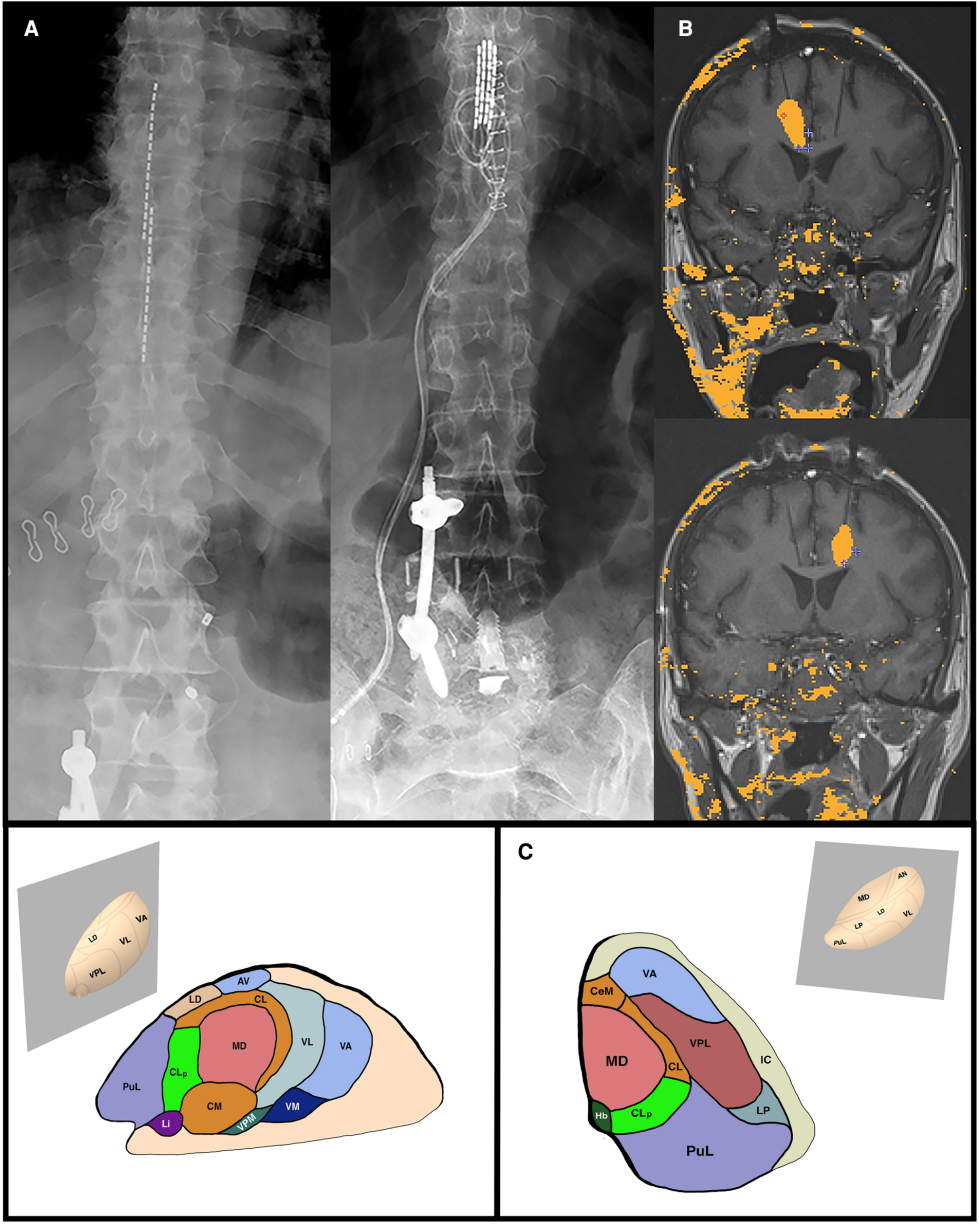
(A) Examples of percutaneous SCS leads (LEFT) and paddle SCS leads (RIGHT). (B) Intraoperative coronal MRI thermometry images of the completed right (TOP) and left (BOTTOM) anterior cingulotomy laser-induced thermal lesions. (C) Thalamic anatomy. Sagittal and Axial planes of the thalamus through the CLp. AV, anterior ventral nucleus; CM, centre median nucleus, CeM, central medial nucleus; CL, central lateral nucleus; CLp, posterior central lateral nucleus; Hb, habenular nucleus; LD, lateral dorsal nucleus; Li, limitans nucleus; LP, lateral posterior nucleus; MD, mediodorsal nucleus; PuL, pulvinar; VA, ventral anterior nucleus; VL, ventral lateral nucleus; VM, ventral medial nucleus; VPM, ventral posterior medial nucleus; VPL, ventral posterior lateral nucleus.

The remaining three papers discuss ablative procedures to manage neuropathic and cancer-related pain. Chalil et al. performed a literature review and illustrated the efficacy of dorsal root entry zone (DREZ) lesioning for brachial plexus avulsion (BPA) injuries in their case series. The authors found that out of 692 patients who underwent DREZ lesioning for BPA injury pain in the literature, 567 were successful in reducing their pain by more than 50%. Furthermore, in a case series of 7 patients, they found that all but one patient achieved more than 50% reduction in pain, with the patients' mean visual analog scale (VAS) score decreasing from 7.9 ± 0.63 pre- to 2.1 ± 0.99 post-operatively. These results confirm pre-existing literature and highlight DREZ lesioning as an efficacious modality to reduce BPA injury pain.

As our understanding of pain has improved, we have learned that pain perception comprises three independent but highly connected components: affective-motivational, sensory-discriminative, and cognitive-evaluative (10). Effective surgical interventions must target one or more of these aspects to reduce pain effectively. While the procedures mentioned above focus on the sensory-discriminative aspect of pain, lesions of cortical and sub-cortical structures target the affective-motivational component of pain. Other ablative targets include the central lateral nucleus of the thalamus and the cingulate cortex. Allam et al. reviewed the role of the central lateral nucleus in processing the affective component of pain and suggested that lesioning of this nucleus may be relatively safe (Figure 1C). In many cases included in the review, patients achieved long-term pain relief even two years after the procedure, with few adverse events reported.

Cingulotomies have traditionally been performed to alleviate widespread pain with a significant psychological component, typically within the head and neck region (11). In this paper, the authors used a relatively novel technique to perform this procedure on a patient with widespread pain due to metastatic cancer. The case report by Allam et al. discussed the ablation of the anterior cingulate cortex *via* MR-guided laser-induced thermal therapy (MRgLITT) in their case report. MRgLITT has only been previously described as a technique for this treatment in five other patients at another institution (Figure 1B) (12–14). While inconclusive, the study supports and illustrates that an MRgLITT cingulotomy can effectively reduce the sensation of widespread cancer pain in appropriately selected patients.

A wide variety of ablative and neuromodulation techniques can be employed for pain management when pharmacologic management has proven inadequate. Most of these procedures, excluding spinal cord stimulation, lack substantial randomized controlled trials and are often used palliatively. Future research is necessary to define the best practices for these procedures to benefit those patients in pain who desire relief from their suffering.

## Author contributions

All authors contributed to the article and approved the submitted version.
